# How the storage effect and the number of temporal niches affect biodiversity in stochastic and seasonal environments

**DOI:** 10.1371/journal.pcbi.1009971

**Published:** 2022-03-28

**Authors:** Immanuel Meyer, Bnaya Steinmetz, Nadav M. Shnerb

**Affiliations:** Department of Physics, Bar-Ilan University, Ramat-Gan, Israel; Abdus Salam International Centre for Theoretical Physics, ITALY

## Abstract

Temporal environmental variations affect diversity in communities of competing populations. In particular, the covariance between competition and environment is known to facilitate invasions of rare species via the storage effect. Here we present a quantitative study of the effects of temporal variations in two-species and in diverse communities. Four scenarios are compared: environmental variations may be either periodic (seasonal) or stochastic, and the dynamics may support the storage effect (global competition) or not (local competition). In two-species communities, coexistence is quantified via the mean time to absorption, and we show that stochastic variations yield shorter persistence time because they allow for rare sequences of bad years. In diverse communities, where the steady-state reflects a colonization-extinction equilibrium, the actual number of temporal niches is shown to play a crucial role. When this number is large, the same trends hold: storage effect and periodic variations increase both species richness and the evenness of the community. Surprisingly, when the number of temporal niches is small global competition acts to decrease species richness and evenness, as it focuses the competition to specific periods, thus increasing the effective fitness differences.

## Author summary

One of the major challenges of community ecology and population genetics is the understanding of the factors that protect biodiversity. Surprisingly, in many generic cases temporal environmental variations (and the abundance fluctuations associated with it) promote the coexistence of competing species and facilitate genetic polymorphism. Here we present a detailed and quantitative comparison between the stabilizing (and the destabilizing) effects of periodic (seasonal) and stochastic temporal variations. When the number of species is small, we show that persistence times under periodic variations are much longer than the persistence times in a stochastic environment. However, environmental variations facilitate coexistence only when the number of temporal niches is larger than the number of species, whereas in the opposite case the same mechanism acts to increase competition and to decrease species richness. Since it is reasonable to expect the number of temporal niches under seasonal variations to be typically smaller than the corresponding number in stochastic environments, stochastic variations provide a more plausible explanation for the apparent stability of high-diversity assemblages.

## I Introduction

Ecological and evolutionary processes take place in varying environments. As a result, demographic rates of populations fluctuate and their abundances vary through time [1]. An increase in demographic variability increases the chance of a population to reach the low-abundance region where extinction is plausible, so environmental variations have the potential to reduce species richness. One of the most surprising and counter-intuitive observations in community ecology has to do with the opposite effect: the ability of temporal environmental variations to *stabilize* a system of competing species and to *increase* the persistence time of diverse assemblages [2–6].

Diverse and even highly diverse communities of competing species are prevalent in nature [7–9], in apparent contradiction with the competitive exclusion principle [10, 11] and/or with the severe limitations exposed by May in his analysis of the complexity-diversity problem [12]. Therefore, the potential role of environmental variations in promoting taxonomic and genetic diversity received a lot of interest. Recently a few theoretical works provided analytical and numerical tools for a quantitative assessment of the stabilizing effect of environmental variations [5, 13, 14], and in parallel, some prominent studies were focused on its manifestation in empirical communities [15–19]. The potential role of stochasticity-induced stabilization in protecting genetic polymorphism was considered as well [20–23].

Although many natural environmental fluctuations are stochastic, most of them have at least some temporal periodicity or seasonality [24, 25]. Quantities like yearly precipitation or the mean yearly temperature indeed fluctuate stochastically, but the amplitude of these stochastic variations is usually much smaller than the amplitude of the variations associated with the seasonal cycle during the year. Seasonal effects on demographic rates and on abundance and frequency variations are well-documented, both for populations [26] and for genotypes [27].

Quite a few works have dealt with the effect of periodic variations on coexistence [24, 28–30]. Here we contribute to this body of work by systematically comparing periodic and stochastic temporal fluctuations with the same characteristics (same amplitude of environmental fluctuations, same mean duration of environment’s dwell time, same level of demographic stochasticity). To do that we would like to implement a set of simple models in which the interpretation of each parameter is transparent. This clarifies the meaning of the results, as well as their relevance to other dynamics.

We consider two types of models. First we deal with two-species communities, where persistence is quantified by the mean time to extinction. This problem has been solved previously for stochastic dynamics [31–33], and here we present solutions for periodic environmental variations and discuss the differences between the cases. Then we analyze diverse communities with extinction-speciation equilibrium, in which case we use species richness and evenness as metrics for the strength of the mechanisms that promote coexistence. Stochastic models of that type, with an unlimited number of niches, were analyzed in [34]; here the study is expanded to include periodic variations and a limited number of temporal niches. All our analyzes are performed for finite communities and demographic stochasticity is explicitly taken into account.

Following former works [31–33], we consider individual-based versions of the lottery model, which is the canonical example of Chesson’s storage effect [2–4]. To solve these models we implement the diffusion approximation, assuming that the characteristic timescales associated with environmental variations are much smaller than the time required for a given population to reach fixation (the “annealed” regime of Mustonen and Lassig [35]). In the opposite (“quenched”) regime, the outcome of a competition is usually decided before an environmental shift occurs, so the role of the environmental variations in protecting coexistence is negligible.

Our study yields two main insights. First, all other things being equal, a community is more stable under periodic variations. Stochastic fluctuations, even when promoting coexistence, may drive a population to extinction through rare sequences of bad years [36]. This scenario is impossible if the variations are periodic. Second, stabilization through the storage effect requires the number of temporal niches to be larger than the number of species. When the number of temporal niches is smaller than the number of species, the same features of the dynamic that yield the storage effect lead to an increase in the effective fitness differences and hence to lower biodiversity.

Seasonal cycles typically involve only a limited number of different states. Therefore, if stochastic variations offer a larger number of temporal niches they will play the main role in stabilizing diverse communities. The emerging picture, and its relevance to practical implications, are summarized in the discussion section.

## II Two-species competition

Two-species competition is the elementary building block of coexistence theory. Here we implement a set of individual-based models, so our theoretical and numerical analyses take into account both demographic stochasticity (stochastic effects that influence the reproductive success of individuals in an uncorrelated manner) and environmental variability. All our models are simple continuous-time (Moran) generalizations of the classical lottery model (see [6] for details).

Global competition allows environmental variations to facilitate coexistence through the storage effect: when an invading given species is superior (produces more seeds, say) its rival, the resident species, is inferior, and therefore the covariance between competition and environment is negative. When competition is local only two individuals compete in each elementary dual, so this covariance disappears. Accordingly, in cases of local competition temporal variations impede coexistence. Here we examine the differences between stochastic and periodic variations in both cases, so overall we consider four scenarios: global-periodic, global-stochastic, local-periodic and local-stochastic.

### II.A Model definitions

We consider a zero-sum competition so the total number of individuals is fixed at *N*. The dynamic takes place in elementary birth-death events, and a generation is defined as *N* such elementary steps. Therefore, the duration of each elementary step is 1/*N*. The (time-dependent) fitness of a given population (species) is *e*^*s*^, so *s* is the log-fitness or the selection parameter.

Due to environmental variations, *s* of each population is time-dependent. In our models, the environment stays fixed for a certain time, which we define as its dwell time *δ*, and then it switches. *δ* is measured in generations, so if *δ* = 1, say, the typical dwell time of the environment is *N* elementary events.

- When the environmental variations are *periodic* or seasonal, the environment switches every *Nδ* elementary steps.
- When the environmental variations are *stochastic*, the chance of the environment to flip after each elementary step is 1/(*Nδ*), therefore, the dwell times are picked from an exponential distribution whose mean is *δ* generations.

Once the value of *s* for each individual is given, it determines its expected reproductive success and its chance to win in a competition. We study two competition scenarios.

- *Local competition* is a characteristic of a community of animals who wander around in a certain spatial region, with an encounter between two individuals resulting in a struggle for a piece of food, territory and so on. To model such a system we consider dynamics that take place via a series of duels between two randomly-picked individuals, in which the loser dies and the winner reproduces. If the fitness of the first individual is exp(*s*_1_) and the fitness of the second is exp(*s*_2_), then the chance of the first to win the competition, *P*_1_, is determined by the fitness ratio
  $$\begin{array}{c} P_{1}=\frac{e^{s_{1}}}{e^{s_{1}}+e^{s_{2}}}. \end{array}$$ (1)
  Analogously, *P*_2_ = 1 − *P*_1_.
- When the competition is global, one individual is chosen at random to die. The chance *P*_*a*_ of an individual *a* to produce an offspring that recruits the resulting open gap is given by
  $$\begin{array}{c} P_{a}=\frac{e^{s_{a}}}{\sum_{b}e^{s_{b}}}, \end{array}$$ (2)
  where the sum ranges over all the individuals. As a result, in the case when all the individuals of the same species have the same fitness, the chance of species 1 (with *n*_1_ individuals) to capture the gap is
  $$\begin{array}{c} P_{1}=\frac{n_{1}e^{s_{1}}}{n_{1}e^{s_{1}}+n_{2}e^{s_{2}}}, \end{array}$$ (3)

Since the fitness of each individual is either *s*_1_ or *s*_2_, Eqs (1) and (3) imply that the outcome of the process depends only on the relative fitness Δ*s* = *s*_1_ − *s*_2_.

Importantly, the storage effect that facilitates invasion of rare populations requires a negative covariance between environment and competition, i.e., when *n*_1_ ≪ *N* the denominator of Eqs 3 or 1 (competition) tends to grow when the numerator (environment) diminishes and vice versa. This never happens in our local competition model, since $\text{Cov}\left[e^{s_{1}^{t}},e^{s_{1}^{t}}+e^{s_{2}^{t}}\right]=\text{Var}\left[e^{s_{1}^{t}}\right]+\text{Cov}\left[e^{s_{1}^{t}},e^{s_{2}^{t}}\right]$. If this expression is indeed negative, then the Cauchy-Schwarz inequality implies that $\text{Cov}[e^{s_{2}^{t}},e^{s_{1}^{t}}+e^{s_{2}^{t}}]$ is positive. Therefore, when the competition is local, environmental variations cannot facilitate the invasion of both species. On the other hand, when the competition is global and *n*_1_, say, is small, the covariance between competition end environment is

$$\text{Cov}\left[e^{s_{1}^{t}},n_{1}e^{s_{1}^{t}}+\left(N-n_{1}\right)e^{s_{2}^{t}}\right]\approx N\text{Cov}\left[e^{s_{1}^{t}},e^{s_{2}^{t}}\right].$$

The same argument holds for the rival species when *n*_1_ ≈ *N* and *n*_2_ ≪ *N*. Therefore, when $\text{Cov}[e^{s_{1}^{t}},e^{s_{2}^{t}}]<0$ the growth rate of each species, when rare, increases due to the storage effect.

As a concrete example, let us consider the case where *x* = *n*_1_/*N* is the frequency of species 1 (that we consider, without loss of generality, as the focal species). The frequency of species 2 (rival species) is 1 − *x*. When species 1 wins, its frequency grows by 1/*N*.

In the local competition model, the chance of an interspecific duel is 2*x*(1 − *x*), so the mean change in *x* after a single elementary event is

$$\begin{array}{c} E\left[x_{t+1/N}-x_{t}\right]=2x\left(1-x\right)\frac{1}{N}(\frac{e^{s_{1}}}{e^{s_{1}}+e^{s_{2}}}-\frac{e^{s_{2}}}{e^{s_{1}}+e^{s_{2}}}). \end{array}$$ (4)

so when |Δ*s*| ≪ 1,

$$\begin{array}{c} \frac{dx}{dt}=x\left(1-x\right)[\left(\Delta s\right)-{\left(\Delta s\right)}^{3}/12+{\left(\Delta s\right)}^{5}/120-\ldots ] \end{array}$$ (5)

The full expansion of *dx*/*dt* in terms of Δ*s* admits only odd powers of Δ*s* multiplied by *x*(1 − *x*). When considering the logit parameter, *z* ≡ ln *x*/(1 − *x*), one notices that it satisfies *dz*/*dt* = (*dx*/*dt*)/[*x*(1 − *x*)]. Therefore, the changes in *z* when Δ*s* is positive are equal in magnitude and opposite in sign to the changes when Δ*s* is negative. As a result, under local competition fitness fluctuations cannot contribute to stability [33, 37, 38], they only lead to an unbiased random walk along the *z* = log[*x*/(1 − *x*)] axis.

The situation changes when competition is global. Now an increase (decrease) in the frequency *x* can happen if one of the rival (focal) species individuals dies [this happens with probability 1 − *x* (*x*)], and the slot is captured by the focal (rival) species. Therefore,

$$\begin{array}{c} E\left[x_{t+1/N}-x_{t}\right]=\frac{x(1-x)}{N}(\frac{e^{s_{1}}}{xe^{s_{1}}+\left(1-x\right)e^{s_{2}}}-\frac{e^{s_{2}}}{xe^{s_{1}}+\left(1-x\right)e^{s_{2}}}). \end{array}$$ (6)

and,

$$\begin{array}{c} \frac{dx}{dt}=\left(\Delta s\right)x\left(1-x\right)+\frac{{\left(\Delta s\right)}^{2}}{2}x\left(1-x\right)\left(1-2x\right)+\ldots \end{array}$$ (7)

Under global competition, one finds in the expansion even terms, like (Δ*s*)^2^, that do not change sign when the mean fitness $s_{0}=\bar{\Delta s}$ changes sign. The coefficient of (Δ*s*)^2^, the term *x*(1 − *x*)(1 − 2*x*), is positive at *x* < 1/2 and negative when *x* > 1/2, so it supports invasion of both species. Therefore, under global competition variations in Δ*s* increase the growth rate of both species when they are rare, and may facilitate a coexistence state.

To complete the picture, we have to quantify the effect of environmental variations, i.e., to specify how the selection parameter varies through time. In our two-species models fitness variations are dichotomous, so Δ*s* is either *s*_0_ + *γ* or *s*_0_ − *γ*. *s*_0_ is the mean fitness difference between the focal and the rival species (i.e., when *s*_0_ is positive the focal species is, on average, superior and vice versa), whereas *γ* is the amplitude of fitness variations. We assume |*s*_0_| ≪ *γ*. A glossary for all parameters is provided in Table 1.

**Table 1:** Glossary.

Term	Description
*N*	number of individuals in the community (all species).
*n*	number of individuals belonging to the focal population.
*x* = *n*/*N*	frequency of focal species (1 − *x* is the fraction of rival species).
*s*_0_	time-independent component of the fitness.
*γ*	the amplitude of fitness fluctuations.
*δ*	dwell time of the environment (measured in generations).
*g* ≡ *δγ*^2^/2	the strength of environmental fluctuations.
$z=\text{ln}(\frac{x}{1-x})$	logit parameter.
*ν*	per-death chance of colonization by new type via speciation, mutation or migration.
*Q*	total number of temporal niches.
*q* = 1…*Q*	temporal niche index.

### II.B Analytic solutions

The main metric for the persistence of a two-species community is *T*, the mean time until one of the species is lost. We will refer to *T* as the time to extinction (of either species).

In our two-species community the state of the system at a given time is fully characterized by the frequency of the focal species *x* and the state of the environment. Since we consider only the annealed dynamics where *x* variations are much slower than environmental variations, we characterize the initial state of the community by *x* alone, so *T*(*x*) is the mean time until extinction, averaged over all initial states of the environment and over all histories [38].

Implementing the diffusion approximation one finds that *T* satisfies [38–40],

$$\begin{array}{c} \frac{\sigma^{2}\left(x\right)}{2}T^{\prime \prime }\left(x\right)+\mu \left(x\right)T^{\prime }\left(x\right)=-1, \end{array}$$ (8)

where *μ*(*x*) is the mean velocity, *σ*^2^(*x*) is the associated variance (see Methods, Section V.A) and prime represent a derivative with respect to *x*. Note that in Eq (8) time is measured in elementary steps, to translate that into generations *T* must be divided by *N*.

In the Methods section we calculate the values of *μ*(*x*) and *σ*^2^(*x*) for four combinations of global (*G*) and local (*L*) competition with stochastic (*S*) and periodic (*P*) environmental variations.

**Local-periodic**: As shown in the previous section, when the competition is local *dx*/*dt* is an odd function of Δ*s*. As explained in the Methods section, this implies that only *s*_0_ contributes to the velocity. Moreover, if the dynamic is periodic *γ* and *δ* have no effect on *σ*^2^(*x*) and *μ*(*x*), which are simply those obtained in the standard case of a fixed environment,

$$\begin{array}{c} \mu_{L,P}\left(x\right)=\frac{s_{0}x\left(1-x\right)}{N}\;\sigma_{L,P}^{2}\left(x\right)=\frac{2x(1-x)}{N^{2}} \end{array}$$ (9)

**Global-periodic**: when the competition is global Eq (7) suggests a bias towards the coexistence point. This bias is independent of the sign of Δ*s* and its strength is proportional to (Δ*s*)^2^, which, when the diffusion approximation holds, may be approximated by *γ*^2^. In the Methods section we found an additional term in the expression for *μ*(*x*). This new term is proportional to *γ*^2^ and represents a bias towards *x* = 1/2.

$$\begin{array}{c} \mu_{G,P}\left(x\right)=\frac{x(1-x)}{N}[s_{0}+\frac{\gamma^{2}}{2}\left(1-2x\right)]\;\sigma_{G,P}^{2}\left(x\right)=\frac{2x(1-x)}{N^{2}}. \end{array}$$ (10)

Since the variations are periodic, they do not contribute to the diffusion term *σ*^2^(*x*).

**Local-stochastic**: stochastic abundance variability increases due to the chance of the focal species, say, to pick a sequence of good or bad years. In the local stochastic case the resulting expressions (Methods, V.A) are (*g* is defined in Table 1),

$$\begin{array}{c} \mu_{L,S}\left(x\right)=\frac{x(1-x)}{N}[s_{0}+g\left(1-2x\right)]\;\sigma_{L,S}^{2}\left(x\right)=\frac{2x(1-x)}{N^{2}}[1+gNx(1-x)]. \end{array}$$ (11)

As before, the *μ*(*x*) has a term that appears to provide a bias towards *x* = 1/2. However, the diffusivity *σ*^2^(*x*) is maximal at *x* = 1/2 and vanishes close to the extinction point *x* = 0 and *x* = 1. The system tends to stick to the regions where its diffusion constant is small [6], and this “diffusive trapping” acts against the stabilizing effect of the *μ* term. These two contradicting tendencies are known to cancel each other exactly [6, 41]. As a result, the only net effect of stochastic environmental variations is an increase in the amplitude of abundance variations, which decreases extinction times [32, 38].

**Global-stochastic**: finally, when competition is global the (Δ*s*)^2^ term facilitates coexistence. As shown in Methods section, the relevant terms are,

$$\begin{array}{c} \mu_{G,S}\left(x\right)=\frac{x(1-x)}{N}[s_{0}+(g+\frac{\gamma^{2}}{2})\left(1-2x\right)]\;\sigma_{G,S}^{2}\left(x\right)=\frac{2x(1-x)}{N^{2}}[1+gNx(1-x)]. \end{array}$$ (12)

While the diffusion term is the same as in Eq (11), the velocity has an extra piece that promotes stability, *γ*^2^*x*(1 − *x*)(1 − 2*x*)/2. This breaks the tie in favor of stabilization and the storage effect manifests itself [33, 38].

**Fig 1:**
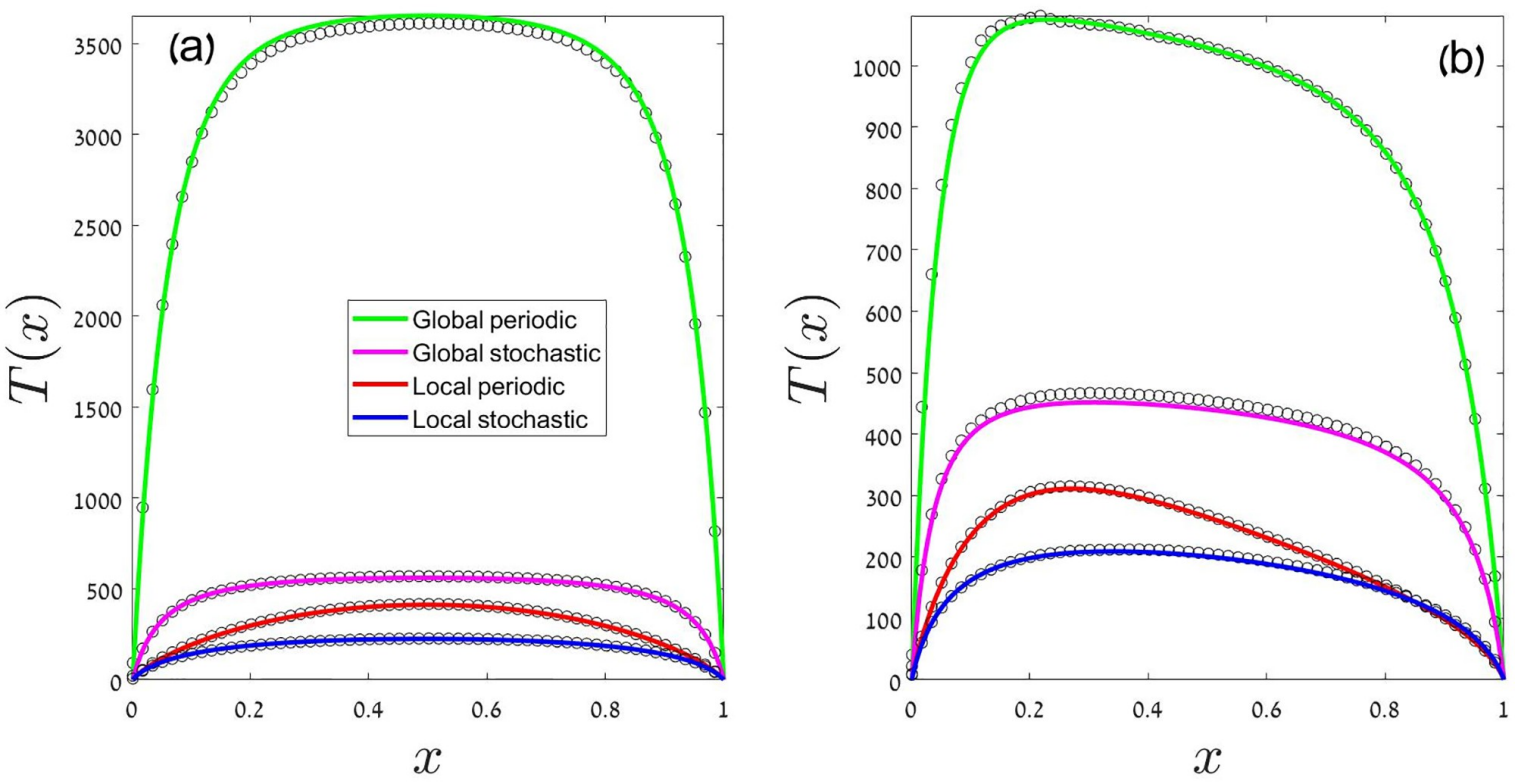
Mean time to extinction, *T*, is plotted against the initial frequency of the focal species *x*. Black circles are the outcomes of a Monte-Carlo simulation, and the colored lines are the theoretical predictions for the corresponding case (see legend) as obtained from numerical solutions of Eq (8) with the *μ*(*x*) and *σ*^2^(*x*) from Eqs (9)–(12). Details of the solution are presented in the Methods section. The hierarchy *T*_*GP*_ > *T*_*GS*_ > *T*_*LP*_ > *T*_*LS*_ is evident. In panel (a), both species have the same mean fitness (*s*_0_ = 0) and therefore all the lines are symmetric around *x* = 1/2. Other parameters in panel (a) are *N* = 600, *γ* = 0.25 and *δ* = 0.55. In panel (b) the focal species is slightly advantageous, with *s*_0_ = 0.01 (all other parameters are the same), so the maximum time to extinction appears at *x* < 1/2.

Eq (8), with the relevant *μ*(*x*) and *σ*^2^(*x*), is an inhomogeneous and linear first order differential equation for *T*′, so one may solve it via an integration factor and then find *T* by an additional integration. When this procedure is implemented numerically, as detailed in the Methods section (V.B), the results fit perfectly the outcomes of our Monte-Carlo simulations, as demonstrated in Fig 1.

Fig 1 reveals the hierarchy of stability properties. The local-stochastic case has the shortest time to extinction, as the role of environmental variations is purely destabilizing. In the local-periodic case this destabilizing effect of environmental variations averages out over each cycle, so its *T* is larger. Under global competition temporal variations facilitate coexistence, so their *T* is larger than in the local cases. Finally, time to extinction in the global-stochastic scenario is shorter than in its global-periodic counterpart: a sequence of bad years may cause extinction in the former, but not in the latter case.

More quantitatively, the mean time to extinction in the local-stochastic and in the global-stochastic cases, and in addition *T* in fixed environment (which, as explained, is equivalent to the local-periodic case), were calculated by [38]. Let us quote some of their result, and contrast them with the new results obtained in the global-periodic case.

When *s*_0_ = 0, or otherwise in the weak selection regime where the effect of *s*_0_ is negligible, the maximum value of *T* (max over all values of initial state *x*) is,

- In the local-stochastic case, *T* ∼ ln^2^ *N*.
- In a fixed environment the maximum value of *T* is linear in *N*. This is an old result, first obtained in [42], and we expect this behavior also in the local-periodic case. Interestingly, [43] obtained this neutral-like results, for both the chance of ultimate fixation and the persistence time, using a model with *global* competition. They consider the parameter regime in which *γ*^2^ is negligible, so the expressions in (10) reduce to those of (9). Deviations from the neutral predictions are then observed only in the quenched regime of [35].
- In the global-stochastic case, *T* ∼ *N*^1/*δ*^.

To understand these different dependencies on *N*, let us neglect the effect of demographic stochasticity and replace it with an absorbing threshold at *z* values that correspond to a single individual, *z* ≈ ±ln *N*. In the local-stochastic case with *s*_0_ = 0 the abundance performs an unbiased random walk along the logit (*z*) axis, therefore exit times scale with ln^2^ *N* [40]. When competition is global and the environment is stochastic the *μ*(*x*) term supports an attractive fixed point in *x* = 1/2, and extinction occurs through improbable sequences of bad years. During such a sequence abundance decreases exponentially, so the number of bad years required to cross the one individual threshold is proportional to ln *N*. Since the chance of such a sequence decreases exponentially with its length, the mean time to extinction scales like a power-law in *N*.

**Fig 2:**
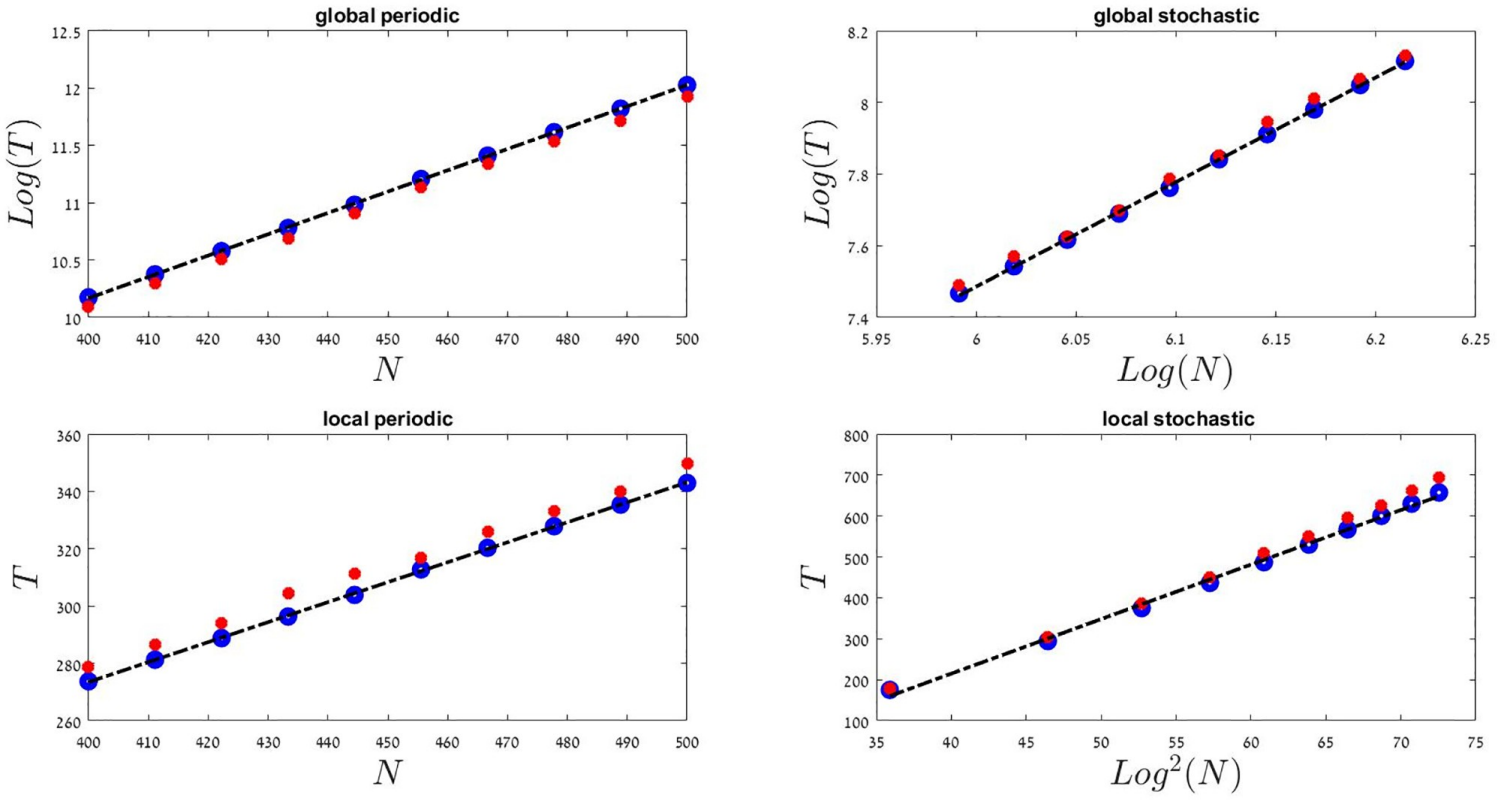
The relationships between the mean time to extinction *T* and the size of the community, *N*, in different scenarios. Filled red circles were obtained from Monte-Carlo simulations, Blue circles are theoretical predictions based on numerical integration of Eq (8) with the *μ*(*x*) and *σ*^2^(*x*) from Eqs (9)–(12), and the dashed black line is a linear fit to these blue circles, presented to guide the eye. As expected, in the global-periodic case the mean time to extinction grows exponentially with *N*, in the global-periodic the grows satisfies a power-law, the local-periodic case behaves like the neutral model (*T* is linear in *N*), whereas the local-stochastic dynamics yields log^2^ *N* growth. In all cases *s*_0_ = 0 (so the *T* shown here is the mean time to extinction starting from *x* = 1/2), *δ* = 0.2 and *γ* = 0.4.

Finally, in the global-periodic case (that was not discussed by [38]) there are no such rare sequences of bad years. Therefore, extinction may take place only due to demographic stochasticity (note that the *σ*^2^(*x*) term in Eq (10) is the same as in Eq (9), reflecting only demographic variations). This requires a highly improbable sequence of death events that may take a population with order *N* individuals to extinction, and such a sequence is exponentially rare in *N*. Therefore, one expects *T* in the global-periodic scenario to grow exponentially with *N*. These four behaviors: ln^2^ *N*, linear, power-law, and exponential, are shown in Fig 2.

When one cannot neglect *s*_0_ the situation is quantitatively different, but the qualitative hierarchy is preserved [38]). In that case under local competition *T* ∼ ln *N* (in both periodic and stochastic cases). When competition is global, as long as |*s*_0_| is not too large and *μ*(*x*) still posses an attractive fixed point at some 0 < *x* < 1, extinction takes place via accumulation of bad years (in the stochastic case) or random death events (in the periodic case). As a result *T* has the same scaling with *N*, although the actual time to extinction for a given set of *N*, *γ* and *δ* become shorter as |*s*_0_| grows.

## III Temporal environmental variations in diverse communities

In the former section, we dealt with a two-species community. When the fluctuations are stochastic a given population may pick a sequence of bad years that will take it to the brink of extinction. Therefore, all other things being equal, coexistence times under seasonal cycles were found to be longer than *T* under stochastic fluctuations. This observation holds for both local competition (no storage effect) and global competition under which environmental variations promote coexistence through storage. Overall, coexistence always benefits from the competition being global and from the environment being periodic.

In this section, we examine the same question in diverse communities, where the steady-state reflects the balance between the rate of extinction and the rate at which new species establish in the community. These new species may reflect immigration from a regional pool in the case of a local community, or may appear due to mutations or speciation events in the course of evolution.

As we shall see below, the number of temporal niches plays a crucial role in the dynamics of diverse communities. In a two-species community, the very assumption of environmental variability presumes the existence of at least two niches: one (say, hotter periods) in which the focal species is doing better and the other (cold periods) when the rival species is advantageous. In communities with fixed speciation or immigration rates, new species always try to invade, therefore the number of species may increase beyond the number of temporal niches.

As the community becomes more and more diverse, every given species evolves under the effect of all other competing species, so the effect of environmental variations is buffered [34]. Therefore, when the number of different species grows, the dynamics become more and more neutral-like. As a result, when the number of temporal niches and the speciation rate are both large, one expects that the community structure will be close to the structure of a corresponding neutral (Hubbell’s mainland model [44, 45], governed by speciation and demographic stochasticity alone) community.

In what follows we would like to emphasize these two effects: the dramatic influence of the number of temporal niches and the neutralization of the dynamics as the diversity of a community grows. To do that, we have simulated all the four dynamics considered above—global and local, periodic and stochastic—in a community exposed to a fixed rate of colonization attempts.

### III.A Models

In our simulations, we implement the same set of competition models defined in Section II.A for the two species case. We add to these models two new parameters. The first, *ν*, reflects the (per-generation) rate in which new types (species, phenotypes) are added to the system via mutation, speciation, or migration from a regional pool. The second parameter, *Q*, is the number of temporal niches.

Every species *i* is endowed with *Q* selection parameters, $s_{i}^{1}\ldots s_{i}^{Q}$, that dictate its fitness in each of the *Q* environments. When a new species arrives (immigration, speciation) a new *s*_*i*_ vector is picked at random. To pick a vector *s*_*i*_ we draw *Q* numbers, ${\tilde{s}}_{i}^{1}\ldots {\tilde{s}}_{i}^{Q}$, from a uniform distribution between −*γ*/2 and *γ*/2, and then defined the *q*-th element of *s*_*i*_ through

$$\begin{array}{c} s_{i}^{q}={\tilde{s}}_{i}^{q}-\frac{1}{Q}\sum_{q=1}^{q=Q}{\tilde{s}}_{i}^{q}. \end{array}$$ (13)

Therefore, all species have the same mean selection parameter, this case is equivalent to the *s*_0_ = 0 case for two-species competition.

Let us describe the elementary timesteps of competition dynamics in a given environment, *q* ∈ [1..*Q*].

If competition is local, two individuals are picked at random for a duel. If one of them belongs to species *i* and the other to species *j*, the chance of the *i* to win the duel is $exp\left(s_{i}^{q}\right)/\left[exp\left(s_{i}^{q}\right)+exp\left(s_{j}^{q}\right)\right]$, for conspecific rivals the chance is 1/2. The winner produces a conspecific offspring that replaces the loser with probability 1 − *ν*. With probability *ν* the loser is replaced by a new type (immigrant, say) that picks a new and random set of *s*^*q*^ values.

If the competition is global, one individual is picked at random to die. With probability *ν* the open gap is recruited by a new type as described above, and with probability 1 − *ν* it is recruited by one of the existing species. The chance of each species *i* to recruit the open gap is given, in parallel with Eq (2) above, by,

$$\begin{array}{c} P_{i}^{q}=\frac{n_{i}e^{s_{i}^{q}}}{\sum_{j}n_{j}e^{s_{j}^{q}}}, \end{array}$$ (14)

where *n*_*j*_ is the abundance of the *j*-th species.

Environmental variations take place as follows. If the system is periodic, then after *δN* elementary timesteps the environment changes from *q* to *q* + 1, and if *q* = *Q* the next environment will be *q* = 1. If the environment is stochastic, after *δN* elementary timesteps we pick an environment *q* at random.

During the simulations, we have monitored the species richness *SR* and the Shannon entropy *SE* after each generation. The Shannon entropy,

$$\begin{array}{c} SE=-\sum_{j=1}^{j=SR}\frac{n_{j}}{N}\text{ln}(\frac{n_{j}}{N}), \end{array}$$ (15)

is a measure of the evenness of the species abundance distribution. It takes the value of zero when there is only one type, and values that are close to zero reflect a community dominated by only one species. For a community with *SR* species, each with abundance *N*/*SR*, *SE* = ln *SR*, therefore, the exponential of *SE* may be considered as the effective number of species [46].

**Fig 3:**
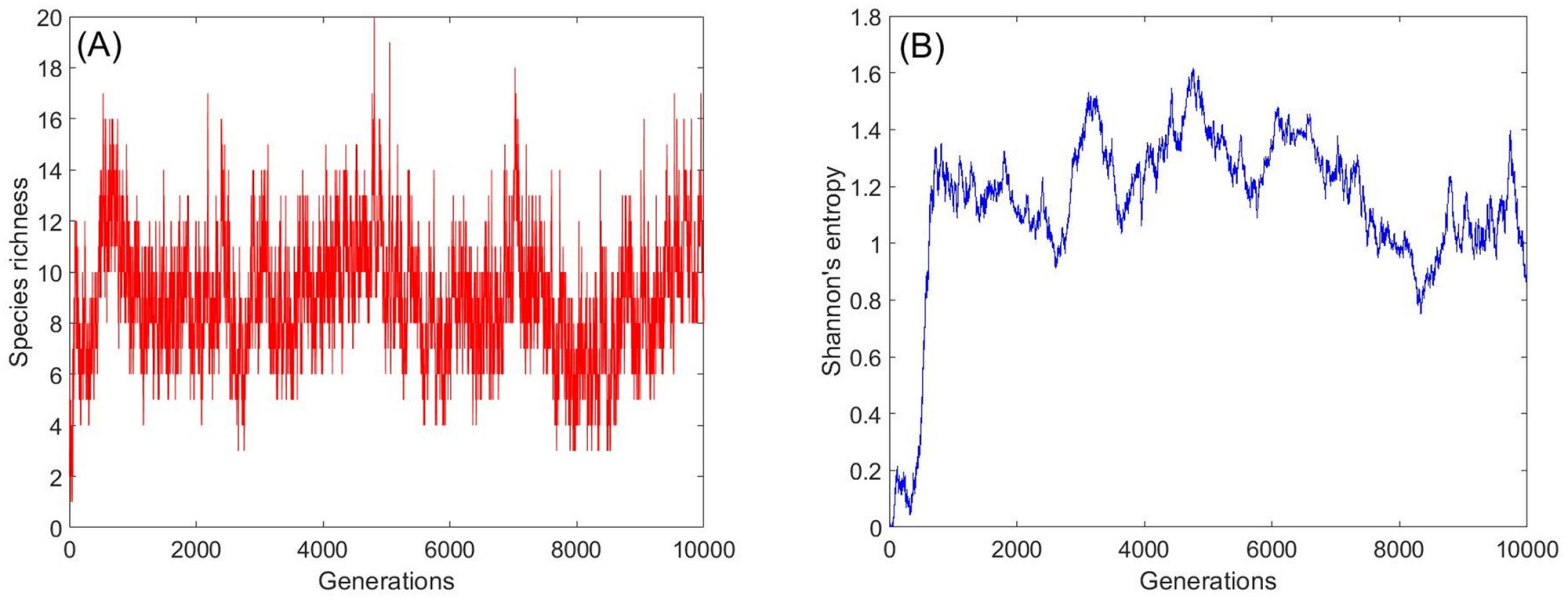
The outcome of a typical simulation. Shown are the species richness (panel A) and Shannon’s entropy (panel B) of a specific run of our simulation. The parameters of this run are *δ* = 0.2, *N* = 10000, *γ* = 0.4 and *ν* = 1/*N*. Competition is global, variations are periodic and the number of niches is *Q* = 3. At the initial state all individuals belong to a single species, hence *SR* = 1 and the entropy is zero. The system equilibrates after less than 1000 generations, and from this point species richness fluctuates around 8.8 and the entropy fluctuates around 1.2.

All simulations started with all individuals belonging to a single species, and we allow the system to equilibrate (to reach the state where both species richness and entropy fluctuate around their typical value). After equilibration we start monitoring the mean and the variance of species richness and entropy, see Fig 3.

### III.B Results

**Fig 4:**
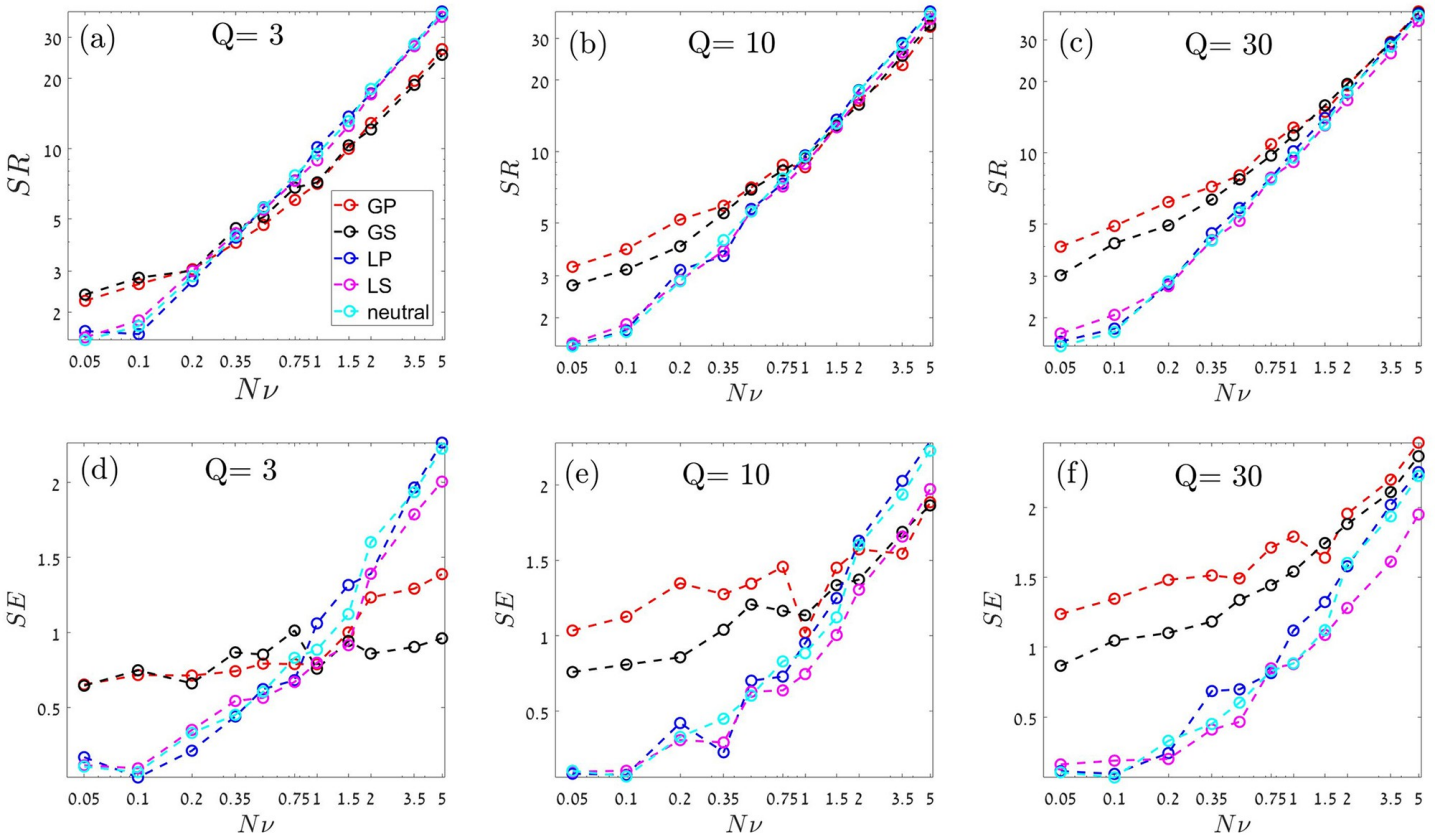
Species richness (*SR*, upper panels) and Shannon’s entropy (*SE*, lower panels) vs. the rate in which new species are trying to invade the community, *νN*. In all cases, when *νN* is small the diversity of a community and its evenness obey the same relationships as *T* in the previous section: global competition is better (for coexistence) than local due to the storage effect, and stochastic variations are worse than periodic variations. When the number of temporal niches is large, as in the *Q* = 30 case in panels (c) and (f), an increase of *νN* leads to an increase in the number of species, their different response buffers the effect of environmental variations and the results converge to the predictions of the neutral model (cyan dashed line). However, as the number of temporal niches decreases global competition puts a hurdle against invasion, as every invader must compete with niche-specialists. Therefore *SR*, and in particular *SE*, are much smaller for global competition if *νN* is large. A comparison between the case of *Q* = 10 [panels (b) and (e)] and *Q* = 3 [panels (a) and (d)] suggest that the crossover from the storage-dominated regime, where global competition is better, to the regime in which global competition acts to decrease biodiversity, occurs when the number of species is equal to the number of temporal niches. For all panels, parameters are: *N* = 10, 000, *δ* = 0.2 and *γ* = 0.4. *SR* and *SE* were monitored every generation and the results presented here reflect an average over the period between 6, 000 and 100, 000 generations. For the neutral model *γ* = 0 and *δ* is an irrelevant parameter.

Fig 4 shows the species richness and the Shannon entropy, as functions of the number of colonization attempts per generation *νN*, for different numbers of temporal niches, *Q* = 3, *Q* = 10 and *Q* = 30.

When the speciation rate *ν* is small, colonization attempts are very rare and the equilibrium species richness is small. In that case, the main factor that dictates community structure is the one considered in the last section, namely the persistence time of a two-species community. When this persistence time is relatively short, an invader species typically goes extinct (or drives the resident species to extinction) before the next colonization attempt, whereas if *T* is large new invaders appear before extinction, and the total species richness increases. Therefore, for all *Q*-s the species richness and the evenness of a community satisfy the relationships of persistence times *T*, as described in the last section: global periodic > global stochastic > local periodic > local stochastic.

As *ν* increases the number of species tends to grow, therefore the number of available temporal niches becomes crucial.

When *Q* = 30, the number of temporal niches is large enough for the community at hand, so it poses no severe constraint on the diversity and evenness of the system. Therefore, what one observes is just the neutralization process: the differential response of many rival species to the environment wipes out the effect of environmental variations. In every given environment each species feels competition from all other individuals and the temporal fluctuations in this quantity (e.g., the mean fitness of a rival individual in local dynamics, or the total number of seeds or larvae in the global dynamics) get smaller in large *SR* [47].

On the other hand, when *Q* = 3 both *SR* and *SE* are much *smaller* when the competition is global. This is a surprising observation: the same phenomenon, global competition, that facilitates coexistence and increases diversity when the number of species is smaller than the number of temporal niches, diminishes the same quantities when the number of temporal niches is smaller than the number of species.

Two arguments may clarify this behavior.

- When the *q*-th niche is still unoccupied, a *q*-specialist (one that has higher fitness in the *q*-environment) benefits from the storage effect when it invades if the competition is global. When the environment favors this invader the competition (total number of seeds or larvae, here $\sum_{j}n_{j}e^{s_{j}^{q}}$) is smaller because the performances of all resident species are worse during that period. Therefore, global competition leads to negative covariance between environment and competition and thus creates the storage effect that promotes invasion. This feature is lost when the competition is local (gains during good years are balanced by losses during bad years). It also weakens significantly when the *q*-invader meets a *q*-resident species that increase its yield, and hence the total competition, during the *q*-periods. As a result, the covariance between competition and environment, and hence the storage effect, weaken significantly when all temporal niches are “occupied”, i.e., when for each temporal niche there is a resident species that specializes in that niche.
- Moreover, under global competition the dynamic of a given species depends more strongly on its fitness when the environment supports it. Therefore, temporal specialists outcompete temporal generalists.
  To understand that, let us consider a simple example. Suppose we have a two-environments-two-species community. Species 1 is a summer specialist, its selection parameter is *s*_1_ = 1 in the summer and *s*_1_ = 0 in the winter. Species 2 is a winter specialist, with *s*_2_ = 1 in the winter and *s*_2_ = 0 during the summer. Assume also that each of these species has *N*/2 individuals when *N* ≫ 1 is the size of the community. Now let us consider the fate of a single individual of species 3. This invading species is a generalist, whose selection parameter is *s*_3_ = 1/2 all year long. The mean (over a full year) of the selection parameter is thus equal for all three species.
  Under local competition the chance of this invader to grow by one after its next interspecific duel is
  $$\frac{1}{2}(\frac{e^{1/2}}{e^{1/2}+1}+\frac{e^{1/2}}{e^{1/2}+e}),$$
  and its chance do decline is
  $$\frac{1}{2}(\frac{1}{e^{1/2}+1}+\frac{e}{e^{1/2}+e}).$$
  These two expressions are equal, so the invader undergoes a neutral dynamics and its invasion growth rate is neither positive nor negative.
  Under global competition, on the other hand, the fraction of seeds of the invader (in both environments) is
  $$f=\frac{2e^{1/2}}{N(1+e)}.$$
  The invader loses one individual with probability (1 − *f*)/*N* ≈ 1/*N*, and gains an individual with probability (1 − 1/*N*)*f* ≈ *f*. Therefore, the chance of the invader to decrease in abundance is higher than its chance to increase and its growth rate is negative.
  Note that in this example both competition and environment are fixed for species 3, so there is no storage effect in any case. Yet, although the mean selection parameter of species 3 is equal to that of species 1 and 2, it becomes inferior under global competition just because, as a generalist, it always loses to the relevant specialist species.

**Fig 5:**
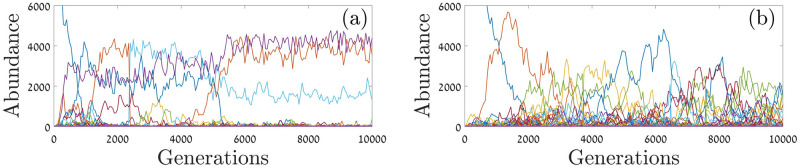
The dynamic of a *Q* = 3 community. The abundance of all species is plotted against time (generations) for global-periodic (left panel) and local-periodic (right panel) competition. Global competition gives an advantage to specialists, therefore the community is dominated by three specialist species, and all other invaders either stay small or (if the invader is a better specialist) take over a given temporal niche and replace the existing specialist. Under local competition, many species coexist, with no gap between the abundances of the dominant species and the abundance of all other species. For both panels, parameters are: *Q* = 3, *N* = 10, 000, *ν* = 0.005, *γ* = 0.4, and *δ* = 0.2.

The differences between global and local competition regimes are further illustrated in Fig 5, where typical histories are plotted through time when the number of temporal niches *Q* is small. As expected, when competition is global the *Q* specialist species dominate the community and avoid colonization by invaders (unless the invader is a better specialist, in which case it replaces the dominant resident that implements the same niche). When the competition is local, temporal niches induce abundance variations but do not select for specialists. Therefore the number of species is higher, and since there is no separation of abundance scales one expects a more even community (higher *SE*).

These results provide us with an interesting, and perhaps general, perspective. When competition is global, the gains of a given species during good years and the losses during bad years are larger than in cases of local competition. This acts in favor of specialists, and in particular generates negative covariance between environment and competition when a given specialist species, whose temporal niche is yet unoccupied, invades a community. On the other hand, once the temporal niche is occupied, the very same feature makes the invasion of other species much harder.

How many distinct temporal niches are allowed in a realistic system? The answer may reflect an essential difference between stochastic fluctuations and seasonal cycles. Environmental stochasticity reflects erratic variations of a wide variety of factors that affect demographic rates—climate, predation pressure, resource availability, diseases, and so on—and the different combinations of these factors suggest, potentially, many temporal niches.

Seasonality, in contrast, is associated in many cases with a limited number of temporal niches. Of course, one may imagine a case in which the maximal fitness of each species appears at the temperature that characterizes a given day of the year, so overall there are 365 temporal niches. However, this possibility requires fine-tuning of the environmental response of different species. In addition, when temporal niches narrow down they become susceptible to stochastic variations. Therefore, periodic temporal niches are usually associated with a coarser partitioning of the environmental states, like the distinction between summer and winter and so on.

## IV Discussion

Temporal niches are ubiquitous in nature, and their dynamics dictate the variations in the fitness of competing species. Some of the main questions in community ecology (and also in population genetics) have to do with the effect of these variations on biodiversity. Through this paper we have analyzed this question in detail, using a variety of models that allow us to isolate the characteristics associated with different features of the dynamics.

An examination of persistence properties in varying environments is not a trivial task. Most studies in that field are based on local analysis: either an analysis of the stability and the robustness of the coexistence states, as implemented, for periodic variations, in [48–51] or an analysis of invasion rates, the standard approach of modern coexistence theory [5, 52]. Here we applied an alternative approach, global analysis, and calculated observable metrics like extinction times, species richness, and evenness of communities, taking into account both demographic and environmental stochasticity. This technique imposed a few restrictions: we had to stick to specific models and use the diffusion approximation and/or numerical simulations. Still, we believe that the outcomes of our study are quite robust and provide a few generic insights.

We discovered that systems with only a few species differ substantially from diverse communities. This distinction has to do with two factors: the number of available temporal niches and the buffering of environmental variations when the number of species is large.

For a system with two species, periodicity and global competition both facilitate coexistence. Therefore, the most prevalent community, with persistence times that grow exponentially with its size *N*, is obtained when competition is global (storage effect) and the dynamic is seasonal. Global competition with stochastic environmental variability is not as prevalent: although it supports storage, rare sequences of bad years may drive a given species to extinction, therefore the mean time to extinction grows like *N*^*α*^ where *α* > 1 is a constant. Local competition does not allow for covariance between environment and competition and thus has no storage. Again, stochastic variations lead to shorter persistence times than the corresponding periodic variations, with persistence times that scale linearly with *N* in the periodic case and with ln^2^ *N* in the stochastic case.

When the number of species increases the situation changes. Most importantly, the number of temporal niches, *Q*, becomes an important limiting factor. The same factors that facilitate storage and support biodiversity when *Q* is larger than the number of species, impede coexistence and lower biodiversity when *Q* is smaller than the number of species. In particular, global competition increases the effect of temporal niches on abundance variations, facilitates the storage effect due to negative covariance between environment and competition, and thus supports the invasion of a specialist into an unoccupied niche. The same feature, global competition, prevents more generalists species to invade an already occupied niche.

Even when the number of temporal niches is large, the differential response of many species buffers the environmental variations and makes the dynamics closer and closer to that of a neutral model, so demographic stochasticity becomes the main driver of abundance variations and temporal environmental fluctuations lose their importance.

Therefore, when environmental variations are invoked as an explanation for the persistence of diverse communities, one must notice that their effect on the community as a whole is not the sum of their effects on each pair of species. A study of many two-species systems, as in the work of [18], for example, may become irrelevant in the diverse community level, either because it misses the neutralization effect or because it does not take properly into account the limited number of temporal niches.

As always, typical natural scenarios are likely to be more complicated. For example, most systems are affected by both stochastic and periodic variations, although the amplitude of periodic variations (in temperature or precipitation between summer and winter, say) is usually much larger than the year-to-year stochastic fluctuations. For example, [24], whose study of plankton community dynamics is based on empirically calibrated parameters, assumed temperature-dependent fitness and modeled temperature variations as a mixture of white noise (with short dwell times) and sinusoidal variations (with long dwell times). This approach is more realistic, but here we have chosen the opposite strategy and compared purely periodic and purely stochastic situations while keeping all other parameters (dwell time, the amplitude of variations, speciation rates, etc.) fixed.

Another scenario that interpolates between stochastic and periodic variations was mentioned by [36]. These authors considered a different stabilizing mechanism, relative nonlinearity, where one species has better fitness but its competitor is more resilient against environmental variations. They have noticed that long periods of stable environment may drive the second species to the brink of extinction, and therefore suggested that the introduction of a low-frequency cutoff of the power spectrum would lead to better coexistence properties. This hypothesis has not been examined in [36], but our study appears to support it.

We thus believe that the results presented in this work provide the essential insights required in assessing the relative importance of temporal niches, their dynamics, and their potential contribution to the richness of life forms in our world.

## V Methods

### V.A Derivation of *μ*(*x*) and *σ*(*x*) in various cases—The diffusion approximation

As explained in the main text, the deterministic change in *x* per unit time during a single step (of duration 1/*N*, *i* is the step index so *t* = *i*/*N*) is,

$$\begin{array}{c} f_{i,local}\left(x\right)\equiv {\dot{x}}_{local}\approx \frac{2x(1-x)}{N}{\left(\Delta s\right)}_{i}, \end{array}$$ (16)

if the competition is local. This equation is equivalent to (5) when |Δ*s*| is small so one may neglect |Δ*s*|^3^ and higher orders.

Equivalently, for global competition Eq (7) implies,

$$\begin{array}{c} f_{i,global}\left(x\right)\equiv {\dot{x}}_{global}\approx \frac{{\left(\Delta s\right)}_{i}x\left(1-x\right)}{N}+\frac{{\left(\Delta s\right)}_{i}^{2}}{2N}x\left(1-x\right)\left(1-2x\right). \end{array}$$ (17)

These two expression define the instantaneous velocity. In addition, every single birth-death event is associated with a variance (demographic fluctuation) of 2*x*(1 − *x*).

In both cases, after *k* steps the mean change in *x* (for a given history of the selection parameter *s*(*t*)) will be,

$$\begin{array}{c} x_{0}\to x_{0}+\sum_{i=1}^{k}f_{i}\left(x_{i}\right), \end{array}$$ (18)

where *f*_*i*_ is the velocity in a given *i* environmental situation (global or local). Expanding this sum to first order around *x*_0_, one gets,

$$\begin{array}{ccc} \sum_{i=1}^{k}f_{i}\left(x_{i}\right) & \approx & \sum_{i=1}^{k}f_{i}\left(x_{0}\right)+\sum_{i=1}^{k}f_{i}^{\prime }\left(x_{0}\right)\left(x_{i}-x_{0}\right)\\ & \approx & \sum_{i=1}^{k}f_{i}\left(x_{0}\right)+\sum_{i=1}^{k}f_{i}^{\prime }\left(x_{0}\right)\sum_{j=1}^{i}f_{j}\left(x_{0}\right). \end{array}$$ (19)

When the environmental fluctuations are periodic, **every** positive period is followed by a negative one of the same amplitude and duration and the internal summation is reduced to order *s*_0_. Once multiplied by the external summation, this term is negligible. Therefore, the mean displacement over several periods is,

$$\begin{array}{c} \mu \left(x_{0}\right)=\frac{\bar{x-x_{0}}}{k}=\bar{f(x_{0})}. \end{array}$$ (20)

Plugging in (16) and (17) yields,

$$\begin{array}{c} \mu_{global,periodic}\left(x\right)=\frac{x(1-x)}{N}[s_{0}+\frac{\gamma^{2}}{2}\left(1-2x\right)]\;\mu_{local,periodic}\left(x\right)=\frac{s_{0}x\left(1-x\right)}{N}. \end{array}$$ (21)

The square of the displacement is,

$$\begin{array}{c} {\left(\sum_{i=1}^{k}f_{i}\left(x_{0}\right)\right)}^{2}=\sum_{i=1}^{k}f_{i}\left(x_{0}\right)\cdot \sum_{j=1}^{k}f_{j}\left(x_{0}\right), \end{array}$$ (22)

and this quantity vanishes, as before, over an integer number of periods. Therefore, the only contribution to *σ*^2^(*x*) comes from demographic stochasticity:

$$\begin{array}{c} \sigma_{global,periodic}^{2}\left(x\right)=\sigma_{local,periodic}^{2}\left(x\right)=\frac{2x(1-x)}{N^{2}} \end{array}$$ (23)

For the stochastic cases we can use the assumption that the environmental states are uncorrelated over periods that are longer than the dwell time, and simplify (19) by grouping together elementary steps into periods in which the environment is fixed,

$$\begin{array}{c} \sum_{i=1}^{k}f_{i}\left(x_{i}\right)\approx \sum_{p}\sum_{q=1}^{\tau_{p}}f_{p}\left(x_{0}\right)+\sum_{p}\sum_{q=1}^{\tau_{p}}f_{p}^{\prime }\left(x_{0}\right)\sum_{j=1}^{q}f_{p}\left(x_{0}\right) \end{array}$$ (24)

In this expression, the *k* elementary steps are divided into periods of stable environments. These periods are indexed by *p*, and the duration of each period is *τ*_*p*_ elementary timesteps. Since the *f*_*p*_ of different *p* are uncorrelated, the internal summation of Eq (19) is reduced to the sum over steps of the same state. Therefore,

$$\begin{array}{c} \sum_{i=1}^{k}f_{i}\left(x_{i}\right)\approx \sum_{p}\tau_{p}f_{p}\left(x_{0}\right)+\sum_{p}f_{p}^{\prime }\left(x_{0}\right)f_{p}\left(x_{0}\right)\frac{\tau_{p}^{2}}{2} \end{array}$$ (25)

When the values of *τ*_*p*_ are drawn from an exponential distribution whose mean is *τ*, $\bar{\tau_{p}^{2}}={\bar{\tau_{p}}}^{2}+\sigma_{\tau }^{2}=2\tau^{2}$, where $\sigma_{\tau }^{2}$ is the variance of *τ*_*p*_. Using,

$$\begin{array}{c} \bar{f^{\prime }\left(x\right)f\left(x\right)}=\frac{1}{2}\bar{\frac{d}{dx}f^{2}\left(x\right)}=\frac{1}{2}\frac{d}{dx}\bar{f^{2}\left(x\right)}\equiv \frac{1}{2}\frac{d}{dx}\sigma_{f}^{2}\left(x\right)=\sigma_{f}^{\prime }\left(x\right)\sigma_{f}\left(x\right). \end{array}$$ (26)

since in the local case, and approximately (neglecting terms like *s*_0_*γ*^2^ or *γ*^4^) in the global case,

$$\begin{array}{c} \sigma_{f}^{2}\left(x\right)\approx \frac{\gamma^{2}x^{2}{\left(1-x\right)}^{2}}{N^{2}}, \end{array}$$ (27)

one can write

$$\begin{array}{c} \mu_{local,stochastic}\left(x\right)=\frac{x(1-x)}{N}[s_{0}+\frac{\gamma^{2}\tau }{2N}\left(1-2x\right)], \end{array}$$ (28)

and

$$\begin{array}{c} \mu_{global,stochastic}\left(x\right)=\frac{x(1-x)}{N}[s_{0}+\frac{\gamma^{2}}{2}\left(1-2x\right)+\frac{\gamma^{2}\tau }{2N}\left(1-2x\right)]. \end{array}$$ (29)

Finally, the variance in the stochastic cases is:

$$\begin{array}{c} \sigma_{global,stochastic}^{2}\left(x\right)=\sigma_{local,stochastic}^{2}\left(x\right)=\frac{2x(1-x)}{N^{2}}[1+\frac{\gamma^{2}\tau }{2}x\left(1-x\right)]. \end{array}$$ (30)

At this point we define *δ*, which is the environmental correlation time measured in generation. If a generation is defined as *N* elementary birth-death events, and on average every *τ* elementary birth-death steps the environment changes, then *δ* = *τ*/*N*. Correspondingly we define *g* = *γ*^2^*δ*/2 as the strength of the environmental variations.

### V.B Solutions for *T* using integration factor

Eq (8) of the main text reads,

$$\begin{array}{c} \frac{\sigma^{2}\left(x\right)}{2}T^{\prime \prime }\left(x\right)+\mu \left(x\right)T^{\prime }\left(x\right)=-1. \end{array}$$ (31)

We solve it by using an integrating factor, such that

$$\begin{array}{c} {\left[T^{\prime }\left(x\right)e^{\int^{x}\frac{2\mu (t)}{\sigma^{2}\left(t\right)}dt}\right]}^{\prime }=-\frac{2}{\sigma^{2}\left(x\right)}e^{\int^{x}\frac{2\mu (t)}{\sigma^{2}\left(t\right)}dt} \end{array}$$ (32)

Integrating on both sides yields

$$\begin{array}{c} T^{\prime }\left(x\right)e^{\int^{x}\frac{2\mu (t)}{\sigma^{2}\left(t\right)dt}}=-\int^{x}\frac{2}{\sigma^{2}\left(t\right)}e^{\int^{t}\frac{2\mu (q)}{\sigma^{2}\left(q\right)}dq}dt+C_{1}\\ T^{\prime }\left(x\right)=-\frac{\int^{x}\frac{2}{\sigma^{2}\left(t\right)}e^{\int^{t}\frac{2\mu (q)}{\sigma^{2}\left(q\right)}dq}dt+C_{1}}{e^{\int^{x}\frac{2\mu (t)}{\sigma^{2}\left(t\right)dt}}}. \end{array}$$ (33)

Finally,

$$\begin{array}{c} T\left(x\right)=-\int^{x}\frac{\int^{k}\frac{2}{\sigma^{2}\left(t\right)}e^{\int^{t}\frac{2\mu (q)}{\sigma^{2}\left(q\right)}dq}dt+C_{1}}{e^{\int^{k}\frac{2\mu (t)}{\sigma^{2}\left(t\right)dt}}}dk+C_{2} \end{array}$$ (34)

All that is left is to find the constants using the boundary conditions *T*(0) = *T*(1) = 0.
